# Empathy Cuts Both Ways in Clinical AI

**DOI:** 10.2196/108778

**Published:** 2026-10-02

**Authors:** Vera Sorin, Eyal Klang

**Affiliations:** 1 Mayo Clinic, Rochester, MN, United States; 2 Beth Israel Deaconess Medical Center, 330 Brookline Avenue, Boston, MA, 02215, United States, 1 617-754-2519

**Keywords:** artificial intelligence, AI, clinical decision support systems, large language models, empathy, explainable AI, trust, human factors, reliance, human-AI interaction

Zhang et al [1] found that participants rated an emotion-adaptive clinical decision support system as clearer, easier to use, more reliable, and more trustworthy than a basic system. Both systems used the same core prediction model and explainability outputs. The empathic system added emotion sensing and a dialogue model that changed how the explanation was delivered.

A clinician who is stressed or confused may understand a difficult explanation better when the system changes how it speaks. But empathy cuts both ways. It can make correct advice easier to accept. It may also make wrong advice harder to reject.

The study measured users’ ratings of trust, reliability, and clarity. It did not test whether the empathic system helped them make more accurate decisions or spot errors. The prediction models were tested separately, but that does not show whether the extra trust was deserved. A system can feel more reliable without being more accurate. A systematic review of large language model empathy found that 11 of 12 studies assessed empathy subjectively [2]. That gap matters because the goal is not the highest possible trust. Trust should match what the system can actually do. Users should accept correct AI advice and reject wrong advice [3].

The authors already identify one part of the problem. Participants reported that emotion adaptation sometimes changed the focus or specificity of the answer. Some replies became overly supportive and did not address the clinical question directly. The authors therefore call for controls that protect clinical precision and decision clarity [1]. But even a direct and precise answer can be wrong. When that answer is also empathic, it may be harder to reject.

This is not only a theoretical concern. In a clinical study, inaccurate advice reduced physicians’ diagnostic accuracy [4]. Another study of health chatbots found that empathic responses increased perceived warmth, trust, and intentions to use the system [5]. These findings do not prove that empathy increases acceptance of wrong clinical advice. They show why the question needs to be tested. One objection is that empathy may raise trust because it improves understanding, not because it persuades. That may be true. The test should separate these effects. Better understanding should help users accept correct advice and reject wrong advice. Persuasion alone may increase acceptance of both.

Future studies should compare correct and wrong AI advice in empathic and neutral language. The clinical content should stay the same. Users could make a judgment before seeing the advice and again afterward. The main outcomes should be final decision accuracy, acceptance of correct advice, rejection of wrong advice, and a direct test of what users understood. Trust ratings can remain, but they should not stand alone.

Emotion-adaptive clinical AI should not make every answer more trusted. It should help trust follow correctness.

## References

[1] Zhang T, Bae SW, Chung T, Dey AK (2026). Emotion-adaptive large language model-driven clinical decision support: user evaluation of the empathic clinical decision support system framework for trust and explainability. JMIR Hum Factors.

[2] Sorin V, Brin D, Barash Y (2024). Large language models and empathy: systematic review. J Med Internet Res.

[3] Schemmer M, Kuehl N, Benz C, Bartos A, Satzger G (2023). IUI ’23: Proceedings of the 28th International Conference on Intelligent User Interfaces.

[4] Gaube S, Suresh H, Raue M (2021). Do as AI say: susceptibility in deployment of clinical decision-aids. NPJ Digit Med.

[5] Seitz L (2024). Artificial empathy in healthcare chatbots: does it feel authentic?. Comput Hum Behav Artif Hum.

